# Preparation of Porous Polymeric Membranes Based on a Pyridine Containing Aromatic Polyether Sulfone

**DOI:** 10.3390/polym11010059

**Published:** 2019-01-02

**Authors:** Nikos D. Koromilas, Charalampos Anastasopoulos, Evdokia K. Oikonomou, Joannis K. Kallitsis

**Affiliations:** 1 Department of Chemistry, University of Patras, GR–26504 Patras, Greece; nikoskoromil@upatras.gr (N.D.K.); xanastasop@upatras.gr (C.A.); 2 FORTH/ICE-HT, Stadiou str., P.O. Box 1414, GR–26504 Rio-Patras, Greece; 3 Laboratoire Matière et Systèmes Complexes, UMR 7057 CNRS Université Denis Diderot Paris-VII, Bâtiment Condorcet, 10 rue Alice Domon et Léonie Duquet, 75205 Paris, France; evdokia.oikonomou@univ-paris-diderot.fr

**Keywords:** polyethersulfone-type membranes, blend, phase inversion, hydrophilicity enhancement, porous membranes

## Abstract

Polymeric membranes, based on a polysulfone-type aromatic polyether matrix, were successfully developed via the non-solvent induced phase separation (NIPS) method. The polyethersulfone type polymer poly[2-(4-(diphenylsulfonyl)-phenoxy)-6-(4-phenoxy) pyridine] (PDSPP) was used as the membrane matrix, and mixed with its sulfonated derivative (SPDSPP) and a polymeric porogen. The SPDPPP was added to impart hydrophilicity, while at the same time maintaining the interactions with the non-sulfonated aromatic polyether forming the membrane matrix. Different techniques were used for the membranes’ properties characterization. The results revealed that the use of the non-sulfonated and sulfonated polymers of the same polymeric backbone, at certain compositions, can lead to membranes with controllable porosity and hydrophilicity.

## 1. Introduction

Water scarcity is one of the most serious complications in modern time [1,2]. The demand for fresh water is predicted to get higher over the next years due to the increase in the world’s population. Water treatment membrane technology has been widely used as a satisfactory approach for the filtration of contaminated water, thus allowing its reuse as clean or drinking water [3]. This increasing interest in the water treatment field has led to the development of inorganic [4] and organic polymeric membranes [5]. Between these two classes, organic polymeric membranes are extensively used due to their ease in processing, low cost, and appropriate robustness.

Rapid growth has been achieved over the last years in the development of water separation processes, including ultrafiltration-microfiltration [6], nanofiltration [7], reverse and forward osmosis [8]. Their use as novel technologies comes up from advantages such as continuous and automatic operation, highly selective separation, little or no need of chemicals, easy scale-up, and low energy consumption. Apart from these advantages, some of the above processes can be combined for increased performance, to achieve sustainable purification of seawater or industrial wastewater.

Filtration membranes can be prepared via various different methods [7], such as interfacial polymerization, phase inversion, post-treatment of a porous support, etc. Phase inversion is considered one of the simplest ones, and can be achieved through non-solvent-induced phase separation (NIPS) [9], evaporation-, vapor-, or thermally-induced phase separation [9]. NIPS has been extensively used for the fabrication of porous membranes based on hydrophobic matrices. Among them, polysulfone (PSF) and polyethersulfone (PES) are used as membrane matrices thanks to their high thermal and chemical stability, mechanical properties, high surface tension, excellent processibility, and film-forming properties [10,11,12,13,14,15]. However, their strong hydrophobic nature makes them prone to fouling, resulting in low water flux and limited lifetime. Therefore, hydrophilicity enhancement of membrane surface is necessary [16], and is key for the membrane function. Nevertheless, rendering these membranes hydrophilic remains a great challenge. To this end, a number of physical or chemical procedures have been used, including the addition of inorganic nanofillers [17] and organic polymers [18]. Regarding the polymers, blending with either water-soluble homopolymers or block copolymers [19] has been attempted. Water-soluble polymers can also be used as pore-forming agents (porogens). The most typical porogens are polyethyleneglycol (PEG) and polyvinylpyrrolidone (PVP), which are incorporated in the membrane by blending with the matrix polymer directly [20], or by using a polysulfone copolymers with PEG, blended with the matrix polymer [21]. Grafting/crosslinking with appropriate polymers [22] is an alternative method that offers stability of the hydrophilic agent. Despite the benefits, the approach of mixing with water-soluble polymers presents a major drawback: The polymers leach out in a high percentage during the phase inversion process due to their hydrosolubility, leading to membrane disruption [23]. Besides, the great majority of hydrophilic polymers are immiscible with membrane matrices, restricting the number of available polymer candidates to very few or demanding more complicated approaches for suppressing immiscibility [24]. 

To overcome these issues in the preparation of membranes with enhanced hydrophilicity, significant efforts have been made by introducing sulfonic acid groups (SO_3_H) into the structure of the hydrophobic polymeric matrix. This can be achieved by blending the polysulfone hydrophobic matrix with a sulfonated polymer [25,26]. These membranes combine all the aforementioned properties of the matrix with the hydrophilicity imparted by the polymer-bearing sulfonic acid groups. The presence of these groups not only increases water flux and water permeability, but also improves the antifouling action [25,26]. Besides, such groups can also offer ion-exchange properties to the membranes [27,28], broadening their application fields.

Based on these scientific results, in this work, blends of the polysulfone-type aromatic polyether [29] poly[2-(4-(diphenylsulfonyl)phenoxy)-6-(4-phenoxy)pyridine] (PDSPP) and its sulfonated derivative (SPDSPP) were synthesized, and porous membranes were formed after precipitation into a non-solvent (water) coagulation bath. Additionally, to better control the morphology of the membranes, water-soluble porogens; like PEG [30] and PVP [31], were incorporated. Blending, as well as the phase inversion mechanism, play a crucial role in the final structure [32]. The effect of various parameters during the whole process interfere in the pore architectures; adjusting the membrane performance [33]. For this reason, the role of different parameters (initial and final total polymers’ concentration, PDSPP/SPDSPP composition, PDSPP molecular weight, type of solvent, time, temperature and composition of coagulation bath, type and concentration of porogen) on the porosity control of these new polysulfone-type aromatic polyethers-based membranes were investigated. As already shown in previous work [34,35], these types of aromatic polyethers—with main and side chain pyridine units—are ideal for membrane synthesis, because they present high thermal and chemical stability, as well as good mechanical properties. The stability of the sulfonated polymer in the membrane was also examined [36]. Here, the sulfonated polymer offers hydrophilicity and does not leach out of the membrane as it is a derivative of the matrix polymer. Finally, the water uptake, flux, and permeability were tested.

## 2. Materials and Methods

### 2.1. Materials

The bis(4-fluorophenyl)sulfone (98%) was purchased from Fluorochem Co., Ltd. (Derbyshire, UK) and used as received. The monomer 2,6-bis(4-hydroxyphenyl)-pyridine was synthesized according to known procedures [37]. The solvents *N*,*N*-dimethylacetamide (DMA, 99%), and *N*-methyl-2-pyrrolidone (NMP, 99+%), were supplied from Alfa Aesar (Ward Hill, MA, USA) and toluene (99.7+%), ethanol (95%), as well as polyethyleneglycol (PEG, MW 2050), polyvinylpyrrolidone (PVP, MW 40,000), and deuterated dimethyl sulfoxide (DMSO-d6) from Sigma-Aldrich Chemical Co., Ltd. (St. Louis, MO, USA) and used without further purification. Solvent 1,1,1-trichloethane (TCE, 97%) was also supplied from Sigma-Aldrich Chemical Co., Ltd. (St. Louis, MO, USA) and distilled prior to use. Chlorosulfonic acid and potassium carbonate were obtained from Merck & Co., Inc (Kenilworth, NJ, USA) and used without further purification. Ultra-pure water was obtained by means of an Ultra Clear TWF UV, SG apparatus water purification unit (SG Wasseraufbereitungund Regenerierstation GmbH, Barsbuettel, Germany).

### 2.2. Instrumentation

Proton Nuclear Magnetic Resonance (^1^H NMR) spectra were obtained on a Bruker Avance DPX 400 spectrometer (Billerica, MA, USA), using d6-DMSO and CDCl_3_ as solvents containing Tetramethylsilane (TMS) internal standard.

Attenuated total reflectance Fourier transform infrared (ATR–FTIR) spectra were recorded on a Platinum ATR Bruker (Billerica, MA, USA). 

Gel Permeation Chromatography (GPC) measurements were carried out at 25 °C with a flow rate of 1 mL/min, using a polymer lab chromatographer equipped with two Ultra Styragel linear columns (10^4^ Å, 500 Å) and a UV detector. Polystyrene standards were used for the calibration and the eluent was CHCl_3_. 

Thermogravimetric analysis (TGA) was performed in alumina crucibles in a Labsys TG apparatus of Setaram (Lyon, France) under nitrogen and at a heating rate of 20 °C/min.

Scanning electron microscopy (SEM) was performed in Zeiss SUPRA 35VP (Oberkochen, Germany) and JEOL 6300 (Tokyo, Japan) instruments equipped with Energy dispersive X-ray spectroscopy (EDS) detectors in order to investigate the top, bottom, and cross-section morphologies of the composite membranes. All membrane samples were frozen in liquid nitrogen and sputtered with gold to produce electric conductivity before SEM examination. In order to investigate the cross section morphology, the samples were broken.

Water uptake was measured according to the dry-wet weight method. Water uptake (WU, %) was calculated as a function of the membrane weight:

(1) $$\mathrm{WU}\left(\%\right)=\frac{W_{w}-W_{d}}{W_{w}}\times 100\%$$

where WU is water uptake of the membrane, *W*_w_ is the wet sample weight (g) and *W*_d_ is the dry sample.

Membrane porosity was measured according to the dry-wet weight method. The porosity (%) was calculated as a function of the membrane weight:

(2) $$\mathrm{Porosity}\left(\%\right)=\frac{W_{w}-W_{d}}{\rho_{w}\;A\delta }\times 100\%$$

where *W*_w_ is the wet sample weight (g), *W*_d_ is the dry sample weight (g), *ρ*_w_ is the density of pure water (g/cm^3^), *A* is the area of membrane in the wet state (cm^2^), and δ is the thickness of membrane in the wet state (cm). 

A vacuum apparatus filtration system was used to measure pure water flux with the effective membrane area of 13.19 cm^2^. The filtration operating was carried out with distilled water at a vacuum pressure of 0.025 MPa. The pure water flux PWF [(L/(m^2^∗h)] was calculated by Equation (3):

(3) $$\mathrm{PWF}=\frac{\Delta V}{A\Delta t}$$

where Δ*V* is the volume of permeated water collected over a period of time (L), A is the effective membrane area for filtration (m^2^), and Δ*t* is the permeation time (h).

Using the same filtration system, pure water permeability PWP [L/(m^2^∗h∗MPa)] was determined, as calculated by Equation (4):

(4) $$\mathrm{PWP}=\frac{\Delta V}{A\Delta t\Delta P}$$

where Δ*V* is the volume of permeated water collected over a period of time (L), *A* is the effective membrane area for filtration (m^2^), Δ*t* is the permeation time (h), and Δ*P* is the membrane pressure (MPa).

### 2.3. Synthesis of Polymers

#### 2.3.1. Poly[2-(4-(diphenylsulfonyl)phenoxy)-6-(4-phenoxy)pyridine] (PDSPP) Synthesis

In a typical reaction, 2,6-bis(4-hydroxyphenyl)-pyridine, bis(4-fluorophenyl)sulfone, and potassium carbonate in equivalent ratios of 1/1/2, respectively, and DMA/toluene in 20/2-fold-excess compared to the monomers, were added to a degassed round bottom flask equipped with a dean stark trap. The mixture was degassed under argon and stirred at 150 °C for 4 h to dehydrate the system. Thereafter, the temperature was increased to 160 °C for 1 h. Then, the temperature was raised topically from the flask to the dean stark trap until total removal of the produced water from the azeotropic mixture toluene-water was achieved. The brownish viscous mixture was cooled down at 25 °C, and precipitated in a 4-fold excess of methanol. After 4 h, the polymer was filtered, washed three times with deionized water and dried at 60 °C under vacuum for 2 days. Polymer synthesis was confirmed by GPC, ATR-FTIR and ^1^H NMR, as shown in Supplementary Information, Figures S1–S3.

#### 2.3.2. Sulfonated Poly[2-(4-(diphenylsulfonyl)phenoxy)-6-(4-phenoxy)pyridine] (SPDSPP) Synthesis

The general procedure is as follows: PDSPP was dissolved in a 30-fold excess of dry TCE in a degassed round bottom flask after stirring for 24 h; then, chlorosulfonic acid (1–3 mol/mol PDSPP) was added dropwise with a dropping funnel at 0 °C in an ice bath for 30 min; afterwards, the ice bath was removed and the mixture was stirred under argon at 25 °C for 24 h; the mixture was then precipitated twice, in a 5-fold excess of 2:1 ethanol/deionised water mixture; and the brownish mixture was filtered, the solid product was thoroughly washed with ethanol and dried at 70 °C under vacuum for 2 days. The sulfonation degree (DS mol %) of the polymer was determined from ^1^H NMR and TGA at 175 and 195 mol %, respectively, as shown in Supplementary Information, Figures S3 and S4.

### 2.4. Preparation of Membranes

#### 2.4.1. Blending

The polymers and hydrophilic polymeric additives were initially dissolved in NMP or DMA in various concentrations and mixed thoroughly under stirring at 25 °C for 24 h until the formation of homogenous solutions at appropriate concentrations. Then, the solutions were removed from the stirring process, to remove microbubble, and poured on a smooth glass substrate at 80 °C, where the solvent was controllably evaporated until the appropriate concentration provided in the Table 1, Table 2, Table 3, Tables 6 and 7 for each system.

#### 2.4.2. Preparation via Phase Inversion

A series of membranes have been prepared with the phase inversion method. When a certain percentage of solvent was reached after casting, the glass plate was immersed in the coagulation bath (water) for immersion precipitation. The prepared membranes were then stored in another water coagulation bath for 24 h to make sure that complete phase separation occurred. The exact experimental conditions (solvent, mixing ratio, temperature) are provided in details in Table 1, Table 2, Table 3 and Table 6. Membranes prepared at different experimental conditions are denoted as Mx-y, where *x* is related to the composition of the PDSPP and SPDSPP. More specifically *x* = 0 refers to 100/0 composition, *x* = 1’ to 90/10 *w*/*w* composition, *x* = 1 to 80/20 *w*/*w* composition and *x* = 2 to 70/30 *w*/*w*. *y* denotes the total concentration of polymers, either the final for the membranes in the Table 1 and Table S3 or the initial for the rest of the membranes. The letters N, D, O and T for the membranes in the preliminary studies are the initials for the words NMP, DMA, overnight, and temperature, respectively. The membranes denoted as Mx,x-y were prepared after immersion in H_2_O/NMP 50/50 *v*/*v* at 25 °C for 1 h instead of H_2_O at 60 °C for 3 h.

For comparison reasons, bulk membranes with no immersion in the coagulation bath were prepared by simple evaporation of the solvent. 

## 3. Results

### 3.1. Synthesis and Characterization of PDSPP and SPDSPP Homopolymers

**Scheme 1:**
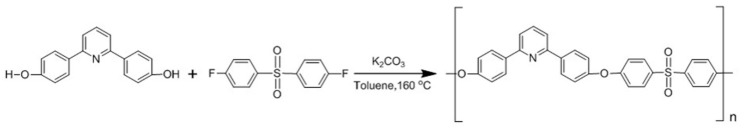
Experimental procedure for PDSPP synthesis.

**Scheme 2:**
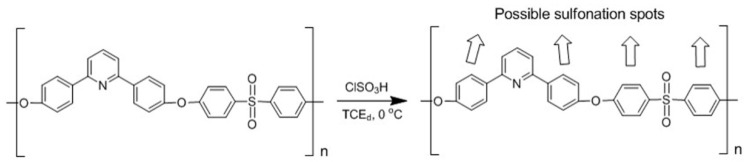
Experimental procedure for SPDSPP synthesis.

The aromatic polyether used for membrane formation was synthesized using the high temperature polycondensation of previously reported diol monomers [29,35]. The specific aromatic polyether was selected for the preparation of membranes, among various analogous functional polysulfone-type polymers, due to its high thermal stability, mechanical strength, chemical inertness, great film-forming properties, ease in high molecular weight synthesis and high sulfonation degrees, which can lead to high water uptake values of its sulfonated derivative. The homopolymer PDSPP was synthesized via step-growth polymerization after the reaction of 2,6-bis(4-hydroxyphenyl)-pyridine and bis(4-fluorophenyl)sulfone in toluene at 160 °C (Scheme 1). The optimization of the reaction conditions led to scale-up synthesis and high molecular weights (Table S1). For this reason, it was possible to handle large quantities of the copolymer either for preparation of membranes with mechanical integrity or for subsequent sulfonation. The latter was achieved with the addition of chlorosulfonic acid in TCE_d_ at 0 °C (Scheme 2). 

For membrane preparation, the homopolymer PDSPP synthesized at high molecular weights was used. The molecular weights were determined by GPC and are exhibited in the Supplementary Information (Figure S1). It is shown that the polymer presented high number-average molecular weight (*M*_n_) and weight-average molecular weight (*M*_w_) values. The polymer used in this work presents a molecular weight (*M*_n_ value) around 50,000.

The chemical structure of the homopolymer PDSPP and its sulfonated derivative SPDSPP was first verified through ATR-FTIR spectroscopy (Supplementary Information, Figure S2). The most characteristic peaks in the PDSPP spectrum at 1236 and 1480 cm^−1^ correspond to the S=O bond of the sulfone and the bond C–N of the pyridine unit, respectively. After sulfonation, a new peak appeared at 1028 cm^−1^ attributed to the symmetrical vibration of SO_3_H units.

The synthesized polymers were also characterized by ^1^H NMR. These results are given in Supplementary Information (Figure S3). In the case of PDSPP homopolymer the presence of aromatic protons was confirmed from the peaks at the region 7.1–8.4 ppm. The degree of sulfonation (DS mol %) was initially determined from the ^1^H NMR spectra (Supplementary Information, Figure S3). Regarding the SPDSPP polymers given here as an example, shift of peaks at over 8.4 ppm was observed due to deprotection of the protons adjacent to the sulfonation spots. The sulfonation percentage was determined by the comparison of the peaks at approximately over 8.4 ppm and 7.1–8.4 ppm. The DS (mol %) of the polymers used for the membrane preparation presented in this work was calculated at 175 mol %.

TGA measurements were also conducted as an alternative qualitative and quantitative characterization method. The results are shown in Supplementary Information, Figure S4, where the thermal decomposition of PDSPP polymer aromatic units started at 400 °C, and was completed at 500 °C, until almost 60% weight loss was observed. On the other hand, SPDSPP polymers display two regions of thermal decomposition, from 274 to 410 °C due to the decomposition of SO_3_H groups [38], and at 410 °C until 600 °C presenting a much higher weight loss in total. Thus, the weight loss in the first area can be used to calculate the sulfonation degree. These results along with the percentage determined from the ^1^H NMR technique are depicted in Supplementary Information, Table S2. There are no significant differences in the results obtained from the two methods. The DS (mol %) from TGA of the membranes used in this work was 195. These results were in agreement with those obtained by ^1^H NMR.

### 3.2. Preliminary Studies for Selection of Conditions for Membrane Preparation

A series of membranes were prepared. At the fist stage, various parameters were explored in order to select the formation conditions providing the optimized membrane porosity. In the first membrane series, 90/10 *w*/*w* PDSPP/SPDSPP dissolved in NMP or DMA at 5% or 15% *w*/*w*, casted at 80 °C until 35%, 50%, 75%, 90% *w*/*w* total final polymers’ concentration and immersed in H_2_O at 25 or 60 °C for 3 h were studied. The list of different tested parameters for the membranes prepared by using NMP are reported in Table 1. The polymers in DMA presented poor miscibility. Hence, the characterization of these membranes is not presented here but the experimental conditions that were tested are exhibited in Supplementary Information, Table S3.

**Figure 1:**
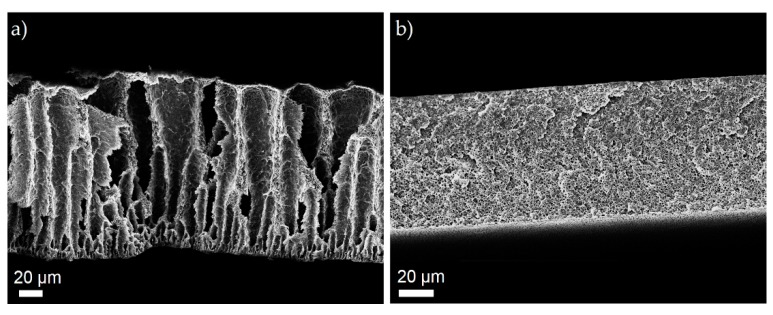
Representative SEM images of the cross-sectional morphologies of the membranes (**a**) M1’-35NT, and (**b**) M1’-75NT.

Initially, the role of the polymer concentration on the cross-section structure was identified. Different concentrations shown in Table 1 were tested. A useful tool to clarify the best formation conditions from all the membranes in NMP proved to be SEM characterization. An example is given in Figure 1, where the cross-sections of the membranes M1’-35NT and M1’-75NT are compared. It is observed that M1’-35NT exhibited an asymmetric structure from a dense skin selective layer at the top to finger-like cavities in the sublayer. The finger-like morphology can be attributed to the relatively low total final concentration (35%, *w*/*w*), the high affinity between NMP and water and the increase of the coagulant temperature at 60 °C resulting in instantaneous demixing during the coagulation phase. On the other hand, the membrane M1’-75NT is dense with sponge-like structure. The explanation for this difference arises from the fact there is a significant increase in the total final concentration (75%, *w*/*w*) leading to higher viscosity of the casting solution, higher resistance of mass transfer during the phase inversion process and eventually, delayed demixing and denser morphology.

Additional parameters were tested. The formation of the membranes at 80/20 *w*/*w* after immersion in H_2_O at 60 °C for 3 h or 24 h (Table 2) provided no additional information regarding the morphology.

**Figure 2:**
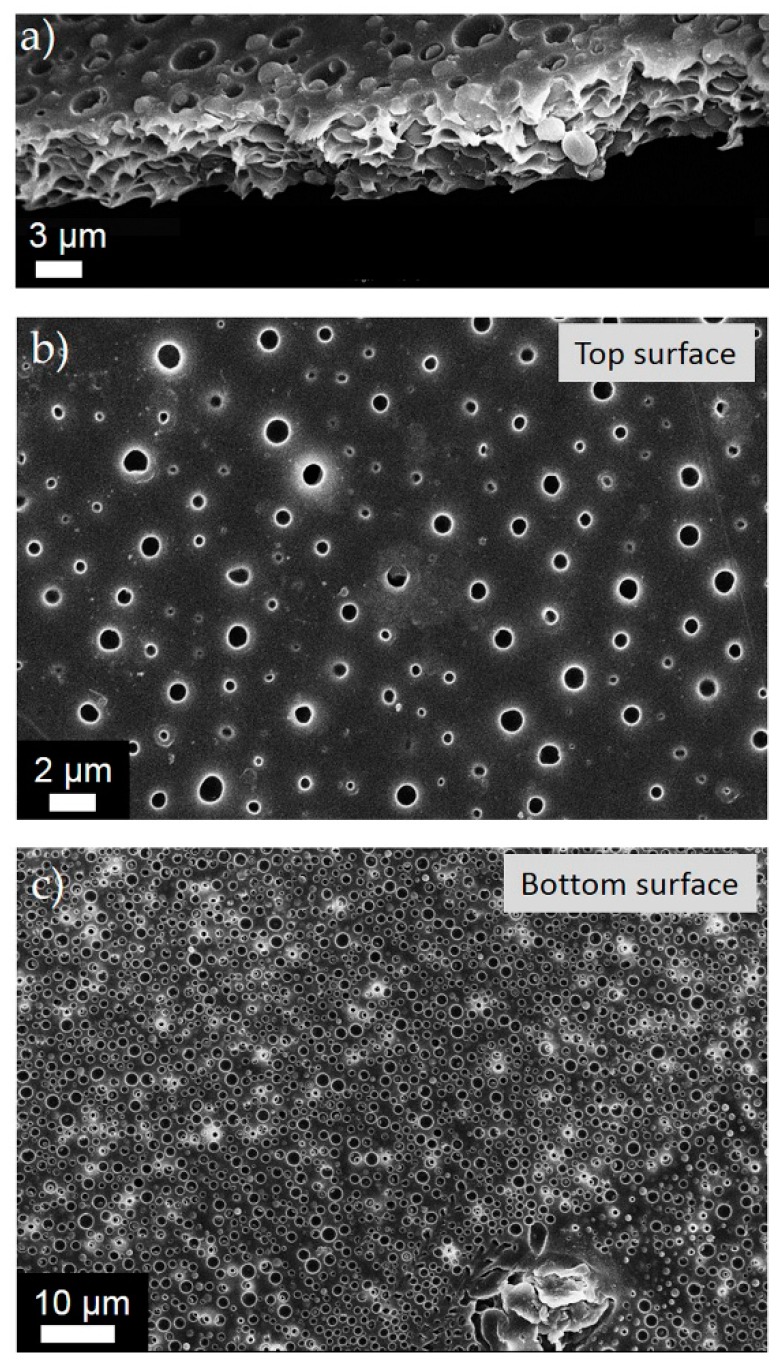
Representative SEM images of (**a**) cross-sectional; (**b**) top; and (**c**) bottom surface morphologies of the membrane M2-PVP6.

To evaluate the role of a porogen on the porous control, the water-soluble polymers poly(vinyl pyrrolidone; PVP) and poly(ethylene glycol) (PEG) were added at 12.5 % *w*/*w* final concentration in a 70/30 *w*/*w* PDSPP/SPDSPP composition (Table 3). It is noteworthy that these membranes formed sponge-like morphology regardless the porogen type confirming the critical role of the high total final concentration (75%, *w*/*w*) in the structure. A higher percentage of pores in the cross section area were obtained after the addition of PVP. The structure of this membrane is given in Figure 2. The pore size for the top and bottom surfaces for the membrane M2-PVP6 were 0.5–1 μm and 1–2 μm, respectively. 

**Figure 3:**
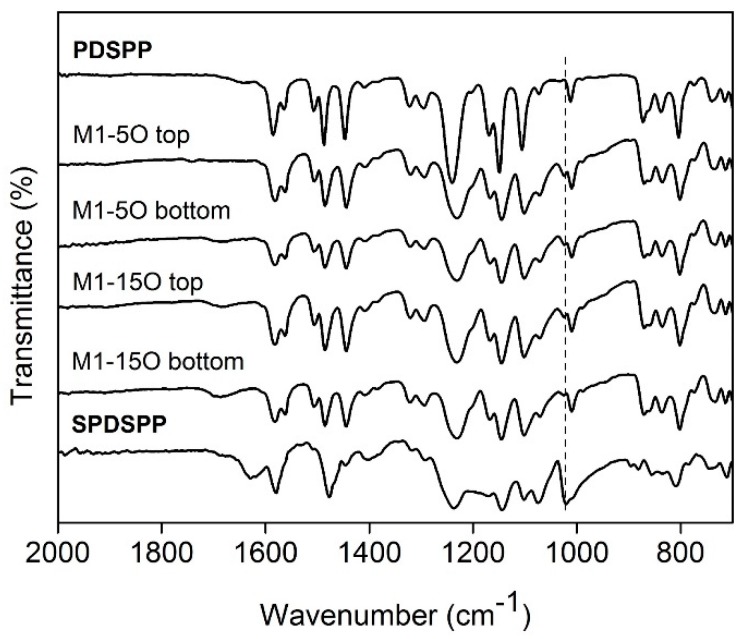
ATR-FTIR spectra of the membranes M1-5O and M1-15O on top and bottom surfaces. For comparison, ATR-FTIR spectra of the PDSPP and SPDSPP homopolymers are shown.

ATR-FTIR was used to verify the existence of the sulfonated polymer in the porous membranes. An example is provided in Figure 3 concerning membranes M1-5O and M1-15O (Table 2). The spectra of the blend membranes resemble mostly with the PDSPP polymer spectrum, displaying the characteristic peak at 1480 cm^−1^. The peak at 1028 cm^−1^ in the SPDSPP spectrum corresponds to the S–O bond of the SO_3_H unit, and appears also in the spectrum of the porous membranes showing the existence of SPDSPP polymer in the membranes. These results prove that SPDSPP polymer exists in the final membranes even after the immersion into the coagulant (water) at 60 °C for 24 h.

Water uptake (WU), pure water flux (PWF) and pure water permeability (PWP) are considered as the key parameters regarding the hydrophilicity, and thus, membrane performance. All these procedures are directly related to porosity. For this reason, the membranes with porous structure were tested. As seen in Table 4, the membranes M1’-35NT, M1’-75NT, M2-PVP6, and M2-PEG6 presented low percentages of water uptake and porosity. Compared to each other, the membrane M2-PEG6 had the higher values of 28.7% and 29.1%, respectively.

Membranes M2-PVP6, M2-PEG6, and M2-PVP18 were selected for the PWF and PWP measurements. The higher values were observed for the membrane M2-PEG6, a result that comes in agreement with the WU and porosity values. In order to evaluate the effect of membrane thickness in PWF and PWP, membrane M2-PVP18 was also tested. Compared with membrane M2-PVP6, it is concluded that the value of membrane thickness is inversely proportional to PWF and PWP values (Table 5). However, membranes with such a low thickness are more susceptible to deformation after a certain point under the operation of constant pressure, so it would be more preferable to use membranes with 12% and 18% initial total concentration that lead to higher thickness values. 

It is noteworthy that the membranes presented low PWF and PWP values in general. These results, even after the addition of water-soluble polymers, suggest that the high final total concentration of polymers affects the porosity due to the low final total concentration of the solvent. As a result, when the coagulant replaces the solvent occupies a small area in the membrane leading to agglomerates. Therefore, it was chosen to reduce the final total concentration of polymers at ≈20% *w*/*w*. 

### 3.3. Morphology of Membranes

Taking into account the aforementioned preliminary results, membranes with the optimum conditions as far as the membrane structure is concerned were prepared. The conditions included dissolution of 80/20 *w*/*w* or 70/30 *w*/*w* PDSPP/SPDSPP in solvent NMP, addition of PVP at 3.3% *w*/*w* final concentration, casting at 80 °C in low final total polymers’ concentrations (≈20% *w*/*w*) and immersion at coagulation bath in H_2_O at 60 °C. At this stage, we chose to formulate membranes at 20% *w*/*w* final total polymers concentration knowing that finger like structures were obtained. This choice was made for better control of membrane porosity, and in a further step this structure would be improved. A series of membranes prepared at these conditions, changing the polymers ratio are presented in Table 6. In the following section, the membranes M1-6 formed with 80/20 *w*/*w* composition and M2-6, M2-12 formed with 70/30 *w*/*w* are discussed. For comparison reasons, the membranes M0-6 and M0-12 with no SPDSPP polymer were also prepared (Table 6). 

**Figure 4:**
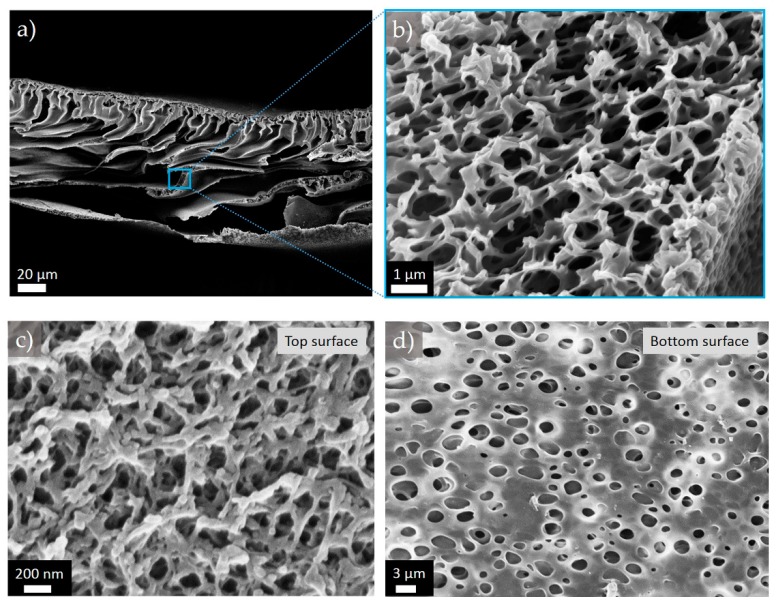
Representative SEM images obtained for the membrane M1-6: (**a**) and (**b**) cross-section at two different magnifications; (**c**) top and (**d**) bottom surface structures.

**Figure 5:**
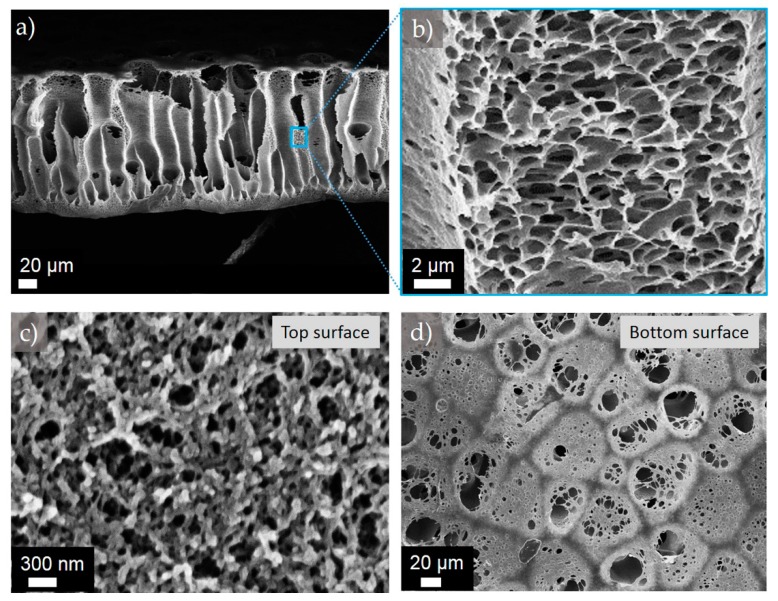
Representative SEM images obtained for the membrane M2-6: (**a**) and (**b**) cross-section at two different magnifications; (**c**) top and (**d**) bottom surface structures.

In Figure 4, SEM images of the cross-section as well as the bottom and top surfaces for membrane M1-6 are depicted. An asymmetric structure with finger-liked macrovoids was observed at the cross-section. However, the porosity in the cross-section (Figure 4b) was very homogeneous with well inter-connected pores in the range 500–800 nm. Concerning the surfaces, the bottom surface exhibited pores with a diameter between 1.5 and 3 μm. In contrast, very small and homogenous porosity was obtained for the top surface (Figure 4c). The pores size is here in the range of 50–100 nm. This homogeneous and small pore structure makes this membrane a good candidate for filtration after improving the finger-like structures. By increasing the SPDSPP content in the blend, membrane M2-6 shown in Figure 5 was obtained. This membrane also presented asymmetric cross-section structures, with a dense layer at the top and cavities located in several spots, as well as high percentage of pores in both surfaces. The cross-section pores were well inter-connected with a pore size in the range of 1–2 µm (Figure 5b). The pores size distribution obtained for the top membrane surface was homogeneous and peaked at 150 nm (Figure 5c), while an inhomogeneous pore structure (0.5–30 µm) was identified for the bottom surface. Both cross-section and top surface porosity were adequate for the water filtration membranes. Both M1-6 and M2-6 presented good porosity showing that the SPDSPP increase did not change significantly the membrane structure. 

**Figure 6:**
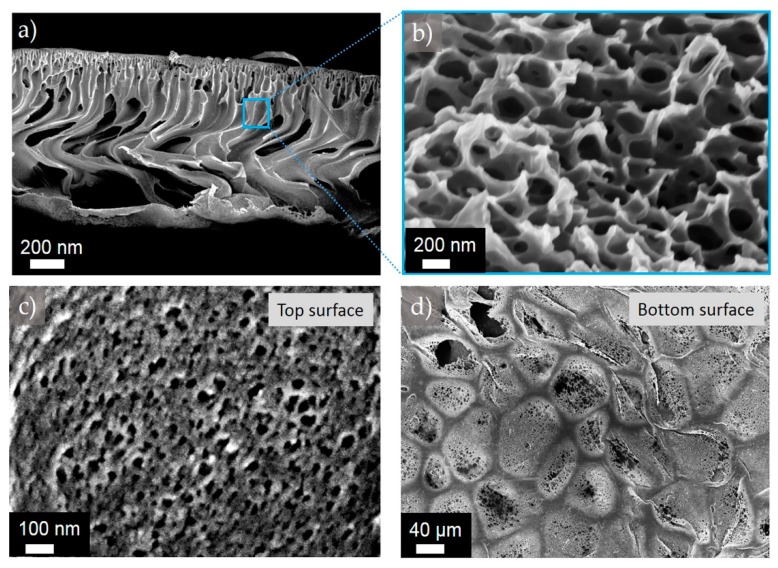
Representative SEM images obtained for the membrane M2-12: (**a**) and (**b**) cross-section at two different magnifications; (**c**) top and (**d**) bottom surface structure.

By increasing the initial total polymer concentration and keeping the other conditions similar to those selected for the M2-6, membrane M2-12 was obtained. The membrane’s structure was similar to the previous ones: Inter-connected homogeneous pores in the cross-section, small pores on the top surface, and large inhomogeneous on the bottom surface. Nevertheless, pore size of both cross-section (~200 nm) and top surface (<100 nm) were much smaller as seen in Figure 6b,c than those obtained for the M1-6 and M2-6 membranes. This makes the membrane a candidate for the nano-filtration process. Besides, concerning the cross-section, the existence of holes and macrovoids was reduced, and better control on cavity formation was achieved. The reduction of demixing time due to the increase in viscosity seemed to affect this membranes morphology [18]. However, the same rule did not apply for membranes M1-6 and M2-12, probably due to the different SPDSPP compositions.

Knowing that the permeation and separation rates of the finger-like were affected from the thickness of the selective skin layer, while the sublayer acted as mechanical support in the membranes with finger-like morphology, the thickness of the skin layer was determined. The value for membrane M2-6 was approximately 12 μm, while it was difficult to estimate the exact thickness for the membrane M1-6. A dense layer skin of 65 μm was obtained for the M2-12. 

**Figure 7:**
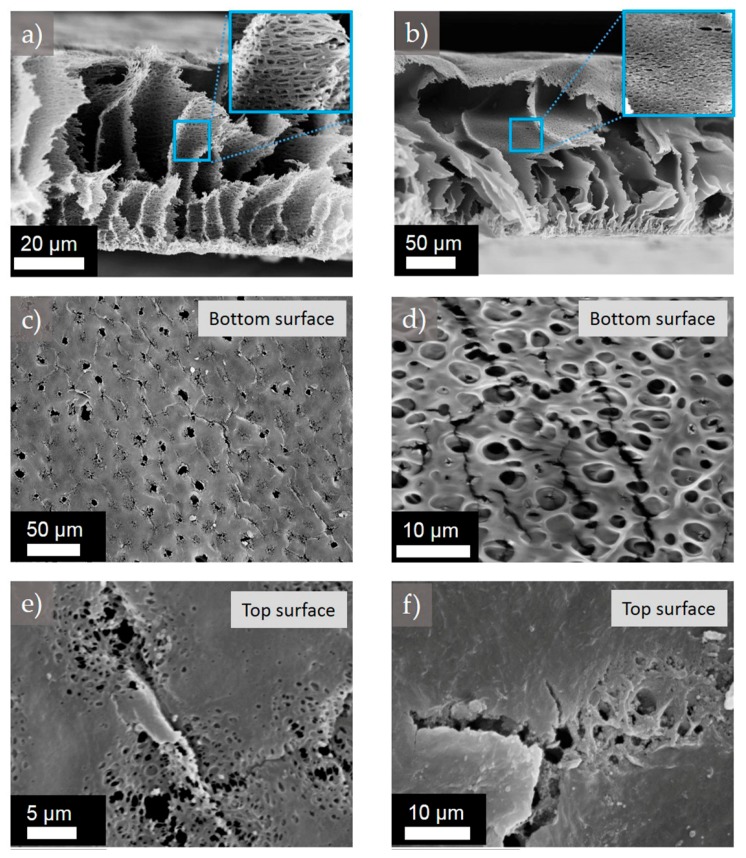
Representative SEM images of the cross-section, the top and the bottom surfaces of the membranes (**a**), (**c**), (**e**) M0-6 and (**b**), (**d**), (**f**) M0-12.

The morphologies of all these membranes deviate from the morphologies of the membranes with no SPDSPP polymer, M0-6, and M0-12 presented in Figure 7a,c,e,b,d,f. Both membranes present finger-like structures with interconnected pores (insets of Figure 7a,b) thanks to porogen use. Nevertheless, in contrast to the above described membranes, the pore sizes and structures were not controlled here. As seen in Figure 7c–f, very large pores were observed on both bottom surfaces (>5 µm), while both top surfaces exhibited dense structures with some uncontrolled micrometric holes. These results reveal that the addition of the sulfonated SPDSPP significantly contributes to the pores formation and control. This can be attributed to interactions between the porogen and the SO_3_H groups that influence the demixing procedure. Hence, SPDSPP not only defines the hydrophilicity of the membranes, but also plays a significant role on the pores’ formation control. 

**Figure 8:**
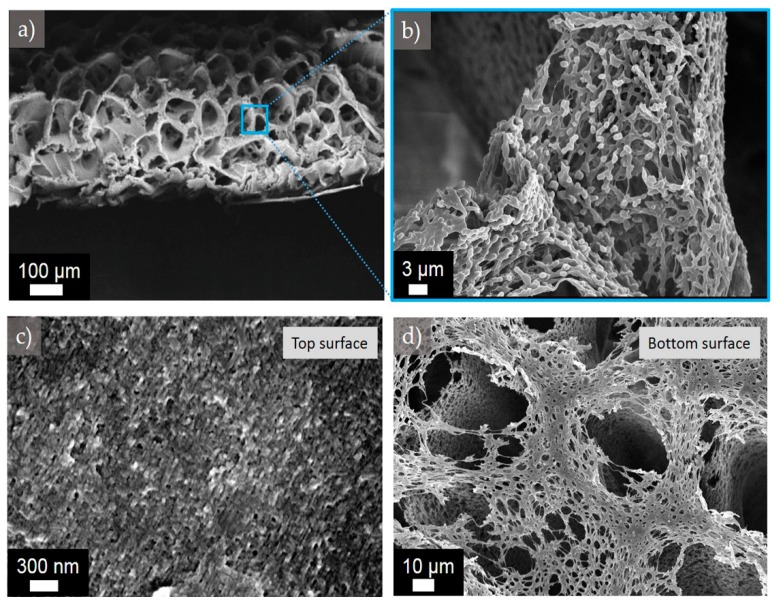
Representative SEM images obtained for the membrane M2-20: (**a**) and (**b**) cross-section at two different magnifications; (**c**) top and (**d**) bottom surface structure.

In order to examine the influence of PVP concentration, membrane M2-20 with a five-fold increase of PVP concentration compared to the membrane M2-12 was prepared. This change altered the morphology from a finger-like to a hive-like connected network, while a large difference in the diameter pores between the two surfaces was obtained (≈50 nm on top, and ≈50 μm on bottom; Figure 8). As mentioned above, PVP is water soluble and is released from the membrane in H_2_O. When this process is well-regulated and takes place at high PVP concentrations, hive-like holes with uniform distribution can be formed.

The studies discussed here show that the combination of the sulfonated polymer with PVP at certain conditions provide membranes with inter-connected pores and controlled small porosity.

**Figure 9:**
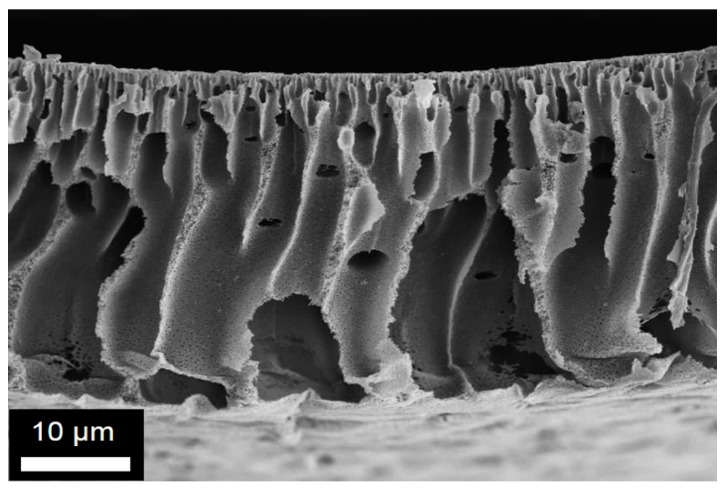
Representative SEM image of the cross-sectional morphology of membrane M2,2-12.

To better improve these membranes as far as the finger like structures are concerned, membranes were prepared using mixtures of H_2_O/NMP 50/50 *v*/*v* as coagulation baths (Table 7). According to the literature, the mixture with the organic solvent can slow down the demixing process giving birth to well controlled sponge-like structures. In Figure 9, we present the cross-section of the membrane with 70/30 *w*/*w* PDSPP/SPDSPP composition, M2,2-12. It is observed that these finger-like structures were restricted compared to membranes M2-6 and M2-12 (Figure 6 and Figure 7), but were not yet completely eliminated. Further studies will be conducted to this direction. 

### 3.4. Characterization of Bulk and Porous Membranes

For evaluating the effect of coagulation in the membranes, and more precisely the alteration in the composition after immersion in water, bulk (not porous) membranes were prepared by simple evaporation of the mixture. This way, the stability of the sulfonated polymer in the matrix after coagulation can be determined. TGA and ATR-FTIR were used as the driving techniques to compare the changes before and after the immersion in the coagulation bath.

**Figure 10:**
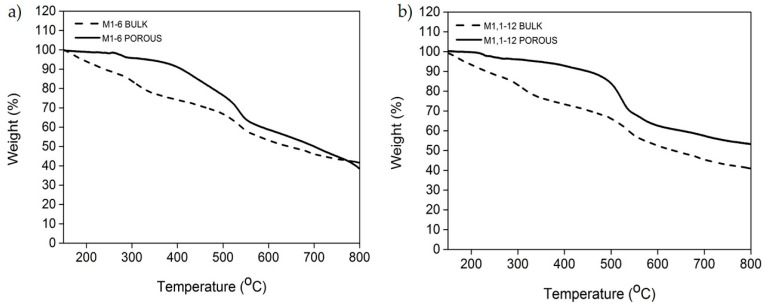
TGA curves of the bulk and porous membranes (**a**) M1-6; and (**b**) M1,1-12.

Characteristic examples of the membranes’ behavior studied by TGA are displayed in Figure 10, where the membranes M1-6 and M1,1-12 present higher thermal stability after coagulation in the region 200 to 500 °C than the same bulk membrane before. Moreover, the two curves resemble mostly the curve observed for the PDSPP homopolymer except the region 200 to 500 °C, where there is a ≈30% weight loss. These findings indicate partial dissolution of PVP homopolymer, because PVP starts to degrade at 400 °C. 

**Figure 11:**
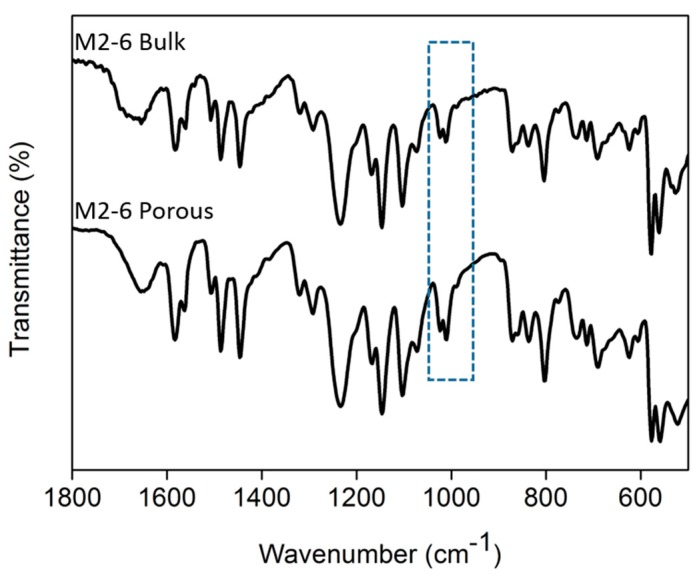
ATR-FTIR spectra of the bulk and porous membrane M2-6.

For further investigation of the changes after coagulation in water, ATR-FTIR characterization was applied. An example concerning the membrane M2-6 is given in Figure 11. It is observed that the spectra of the bulk and porous membrane M2-6 were similar, although a reduction in the peak at 1660 cm^−1^ was observed. More specifically, the characteristic peaks at 1028, 1440, and 1660 cm^−1^ correspond to the symmetrical vibration of SO_3_H units of SPDSPP, the C–N bond of SPDSPP, and the C=O bending of PVP, although the latter overlapped by the SPDSPP peak. Therefore, the maintenance of the peak at 1028 cm^−1^ proves the existence of SPDSPP polymer in the porous membrane after coagulation in water. This means that the polymer was not solubilized during coagulation, indicating that it would not leach out during filtration process. The peak at 1290 cm^−1^ corresponding to the C–N bond of PVP [39] overlapped with a PDSPP peak. As for the reduction of the peak at 1660 cm^−1^ in the porous membrane, it was due to the partial dissolution of PVP after coagulation. The PVP partial dissolution was not unlikely to happen, because the nitrogen of the homopolymer formed hydrogen bonds with the SO_3_H unit of the SPDSPP homopolymer resulting at a certain degree in the suppression of the leaching process through the membrane in the coagulation bath. 

### 3.5. Hydrophilicity and Porosity Measurements

**Figure 12:**
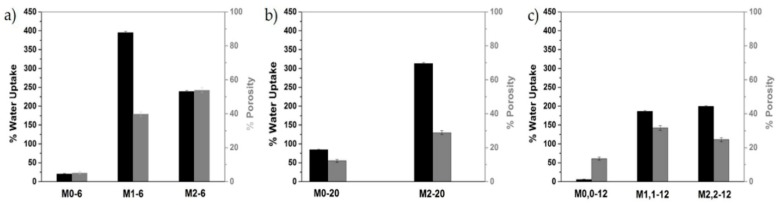
Schematic representation of WU and porosity values for the membranes (**a**) M0-6, M1-6, and M2-6; (**b**) M0-20, and M2-20; and (**c**) M0,0-12, M1,1-12, and M2,2-12.

The water uptake (WU) and porosity measurements for membranes M0-6 and M1-6, M2-6 (Table 8) were consistent with the SEM micrographs and the formation conditions. Thus, the membranes M1-6 and M2-6 had much higher values compared to the corresponding membranes without the sulfonated polymer, M0-6 (Figure 12a) and between them, membrane M2-6 presented the highest porosity value (53.9%) and membrane M1-6 the highest WU value (395.2%). In general, the porosity and WU values were relatively close for these membranes. The only contrast in these results seems to be the WU value of 239.2% for membrane M2-6, which cannot be explained from the experimental procedure.

After the increase of PVP concentration, the measurements for membrane M2-20 revealed the expected high WU value at 313.3% and medium porosity value at 28.8% (Figure 12b). The porosity value was also reasonable, as the porosity was directly related to the morphology of the membrane, as described in the SEM images. The calculations regarding the membranes M0,0-12 and M1,1-12, M2,2-12 followed the same route as there was significant increase in the WU and porosity values for the membranes M1,1-12, M2,2-12 compared to the membrane M0,0-12 (Figure 12c). 

These high values for both WU and porosity were not so common for the polymeric membranes, because high hydrophilicity resulted in an increase in the membrane swelling that might shrink the effective pore volume and lower the membrane porosity [40].

### 3.6. Filtration Tests

Membranes M2-6 and M2-12 were evaluated for their PWF and PWP performance. The membranes were chosen due to their controlled porosity and the similarity in formation conditions. As shown in Table 9, the membranes exhibited very high PWF and PWP values compared to membranes M1-PVP6, M3-PEG6, and M1-PVP18. Among them, membrane M2-12 presented the higher PWF and PWP values of 1820.2 L/(m^2^∗h) and 72794.2 L/(m^2^∗h∗MPa), respectively. The results revealed that the control of formation conditions leads to great membrane performance regarding the water filtration. 

## 4. Conclusions

Porous membranes based on synthesized pyridine-based aromatic polyether (PDSPP) and its sulfonated analogue (SPDSPP) were prepared by a non-solvent inversion technique. The combination of high MW aromatic polyether with its hydrophilic sulfonated counterpart enabled both the good miscibility between the hydrophobic and hydrophilic polymers, and the hydrophilic modification of the final membrane. After an initial study of the structural porous properties, the best compositions were selected in order to prepare and characterize the final membranes. The membranes prepared under these conditions exhibited finger-like structures and well inter-connected pores in the cross-section whose size varied from 200 nm to 2 µm depending on the preparation conditions. The surface pores were very homogeneous and their size was in the range of 100–200 nm on the top surface, making them good candidates for water purification. It was shown that in absence of the SPDSPP, the obtained porosity was uncontrolled and not homogeneous. These observations reveal that the SPDSPP not only offers hydrophilicity but also plays a crucial role in determining pore structure and size. Some of the membranes exhibited high WU and porosity values, and high PWF and PWP values calculated from the pure water filtration tests. The stability of the hydrophilic agent; SPDSPP, in the membrane after the coagulation process was ascertained by ATR and shown to be stable probably thanks to its compatibility with the matrix polymer. Given that the synthesis of these polymers was well controlled, several different parameters such as the molecular weight and the sulfonation degree of the SPDSPP can be further tested for optimizing membrane structure according to the envisaged application.

## Figures and Tables

**Table 1:** Preparation of membranes with 90/10 w/w PDSPP/SPDSPP in NMP at 5% or 15% *w*/*w*, casting at 80 °C until 35%, 50%, 75%, 90% *w*/*w* total final polymers’ concentration and immersion in H_2_O at 25 °C or 60 °C for 3 h.

Membrane	PDSPP Initial/Final Concentration (*w*/*w* %)	SPDSPP Initial/Final Concentration (*w*/*w* %)	Initial/Final Concentration (*w*/*w* %) in NMP	Temperature (°C)/Coagulation Time (h) in H_2_O	Membrane Thickness (μm)
M1’-35N	13.5/31.5	1.5/3.5	85/65	25/3	200
M1’-50N	13.5/45	1.5/5	85/50	25/3	220
M1’-75N	13.5/67.5	1.5/7.5	85/25	25/3	50
M1’-90N	13.5/81	1.5/9	85/10	25/3	45
M1’-35NT	13.5/31.5	1.5/3.5	85/65	60/3	350
M1’-50NT	13.5/45	1.5/5	85/50	60/3	290
M1’-75NT	13.5/67.5	1.5/7.5	85/25	60/3	90
M1’-90NT	13.5/81	1.5/9	85/10	60/3	70

**Table 2:** Preparation of membranes with 80/20 *w*/*w* PDSPP/SPDSPP in NMP at 5% or 15% *w*/*w*, casting at 80 °C until 75% *w*/*w* total final polymers’ concentration and immersion in H_2_O at 60 °C for 3 h or 24 h.

Membrane	PDSPP Initial/Final Concentration (*w*/*w* %)	SPDSPP Initial/Final Concentration (*w*/*w* %)	Initial/Final Concentration (*w*/*w* %) in NMP	Temperature (°C)/Coagulation Time (h) in H_2_O	Membrane Thickness (μm)
M1-5O	4/60	1/15	95/25	60/24	20
M1-5	4/60	1/15	95/25	60/3	20
M1-15O	12/60	3/15	85/25	60/24	85
M1-15	12/60	3/15	85/25	60/3	90

**Table 3:** Preparation of membranes with 70/30 *w*/*w* PDSPP/SPDSPP in NMP at 5% or 15% *w*/*w*, addition of PVP or PEG at 12.5% *w*/*w* final concentration, casting at 80 °C until 75% *w*/*w* total final polymers’ concentration and immersion in H_2_O at 60 °C for 3 h.

Membrane	PDSPP Initial/Final Concentration (*w*/*w* %)	SPDSPP Initial/Final Concentration (*w*/*w* %)	Initial/Final Concentration (*w*/*w* %) in NMP	Porogen Initial/Final Concentration (*w*/*w* %)	Temperature (°C)/Coagulation Time (h) in H_2_O	Membrane Thickness (μm)
M0-5	3.5/52.5	1.5/22.5	95/25	-	60/3	20
M0-15	10.5/52.5	4.5/22.5	85/25	-	60/3	60
M2-PVP6	3.5/43.7	1.5/18.8	94/25	1/12.5	60/3	15
M2-PVP18	10.5/43.7	4.5/78.8	82/25	3/12.5	60/3	80
M2-PEG6	3.5/43.7	1.5/18.8	94/25	1/12.5	60/3	25
M2-PEG18	10.5/43.7	4.5/18.8	82/25	3/12.5	60/3	55

**Table 4:** WU and porosity values for the membranes M1’-35NT, M1’-75NT, M2-PVP6, and M2-PEG6.

Membrane	WU (%)	Porosity (%)
M1’-35NT	12.7 ± 0.8	4.3 ± 0.5
M1’-75NT	13.4 ± 1.0	15.2 ± 0.9
M2-PVP6	21.2 ± 1.3	21.0 ± 1.2
M2-PEG6	28.7 ± 1.1	29.1 ± 1.3

**Table 5:** PWF and PWP values for the membranes M2-PVP6, M2-PEG6, and M2-PVP18.

Membrane	PWF [L/(m^2^∗h)]	PWP [L/(m^2^∗h∗MPa)]
M2-PVP6	12.1 ± 2.2	50 ± 3.2
M2-PEG6	82.4 ± 3.6	3300 ± 9.1
M2-PVP18	0.2 ± 0.1	8.7 ± 1.6

**Table 6:** Preparation of membranes with 80/20 *w*/*w* or 70/30 *w*/*w* PDSPP/SPDSPP in NMP at 5% and 15% *w*/*w*, addition of PVP at 3.3% *w*/*w* final concentration, casting at 80 °C until 20% *w*/*w* total final polymers’ concentration and immersion in H_2_O at 60 °C for 3 h.

Membrane	PDSPP Initial/Final Concentration (*w*/*w* %)	SPDSPP Initial/Final Concentration (*w*/*w* %)	Initial/Final Concentration (*w*/*w* %) in NMP	PVP Initial/Final Concentration (*w*/*w* %)	Temperature (°C)/Coagulation Time (h) in H_2_O	Membrane Thickness (μm)
M0-6	5/16.7	-	94/80	1/3.3	60/3	50
M1-6	4/13.3	1/3.3	94/80	1/3.3	60/3	70
M2-6	3.5/11.7	1.5/5	94/80	1/3.3	60/3	70
M0-12	10/16.7	-	88/80	2/3.3	60/3	170
M2-12	7/11.7	3/5	88/80	2/3.3	60/3	280
Μ2-20	7/7	3/3	80/80	10/10	60/3	265

**Table 7:** Preparation of membranes with 80/20 *w*/*w* or 70/30 *w*/*w* PDSPP/SPDSPP in NMP at 10%, addition of PVP at 3.3% *w*/*w* final concentration, casting at 80 °C until 20% *w*/*w* total final polymers’ concentration and immersion in H_2_O/NMP 50/50 *v*/*v* at 25 °C for 1 h.

Membrane	PDSPP Initial/Final Concentration (*w*/*w* %)	SPDSPP Initial/Final Concentration (*w*/*w* %)	Initial/Final Concentration (*w*/*w* %) in NMP	PVP Initial/Final Concentration (*w*/*w* %)	Temperature (°C)/Coagulation Time (h) in H_2_O/NMP 50/50 v/v	Membrane Thickness (μm)
M0,0-12	10 /16.7	-	88/80	2/3.3	25/1	80
M1,1-12	8/13.3	2/3.3	88/80	2/3.3	25/1	180
M2,2-12	7/11.7	3/5	88/80	2/3.3	25/1	260

**Table 8:** WU and porosity values for membranes M0-6, M1-6, M2-6, M0,0-12, M1,1-12, and M2,2-12.

Membrane	WU (%)	Porosity (%)
M0-6	20.5 ± 1.2	5.1 ± 0.8
M1-6	395.2 ± 2.9	39.8 ± 1.4
M2-6	239.2 ± 2.4	53.9 ± 1.5
M0-20	84.6 ± 1.1	12.2 ± 0.8
M2-20	313.3 ± 2.3	28.8 ± 1.2
M0,0-12	5.2 ± 0.8	13.5 ± 0.9
M1,1-12	186.4 ± 2.3	31.6 ± 1.4
M2,2-12	199.6 ± 2.1	24.7 ± 1.2

**Table 9:** PWF and PWP values for membranes M2-6 and M2-12.

Membrane	PWF [(L/(m^2^∗h)]	PWP [L/(m^2^∗h∗MPa)]
M2-6	827.1 ± 10.6	33084.7 ± 36.2
M2-12	1820.2 ± 11.5	72794.2 ± 37.8

## Supplementary Materials

The following are available online at http://www.mdpi.com/2073-4360/11/1/59/s1, Figure S1: GPC chromatograph of the PDSPP homopolymer; Figure S2: ATR-FTIR spectra of the PDSPP and SPDSPP homopolymers; Figure S3: ^1^H NMR spectra of the PDSPP and SPDSPP homopolymers; Figure S4: TGA curves of the PDSPP and SPDSPP homopolymers; Table S1: Quantities, along with Mn, Mw, and PDI as calculated from the GPC characterization for the PDSPP homopolymers; Table S2: DS (mol%) estimated from the ^1^H NMR and TGA techniques, as well as IEC for the S1PDSPP and S2PDSPP homopolymers; Table S3: Preparation of membranes with 90/10 *w*/*w* PDSPP/SPDSPP in DMA, at 5% or 15% *w*/*w*, casting at 80 °C until 35%, 50%, 75%, 90% *w*/*w* total final polymers’ concentration and immersion in H_2_O at 25 °C or 60 °C for 3 h.
