# Supplementary material for: Preparation of Porous Polymeric Membranes Based on a Pyridine Containing Aromatic Polyether Sulfone
Source: Polymers (Basel). 2019 Jan 2;11(1):59. doi: 10.3390/polym11010059 (PMC6402226; doi:10.3390/polym11010059)
Supplement: Supplementary file 1 [file polymers-11-00059-s001.zip › polymers-403298-Supplementary Materials/Supplementary Information_Polymers.docx]

Supplementary Information

Preparation of porous polymeric membranes based on a pyridine containing aromatic polyether sulfone

Nikos D. Koromilas ^1,2^, Charalampos Anastasopoulos ^1^ Evdokia K. Oikonomou^3^ and Joannis K. Kallitsis ^1,2,^*

^1^ Department of Chemistry, University of Patras, GR–26504, Patras, Greece; [xanastasops@upatras.gr](mailto:xanastasops@upatras.gr) (C.A.)

^2^ FORTH/ICE-HT, Stadiou str., P.O. Box 1414, GR–26504, Rio-Patras, Greece; nikoskoromil@upatras.gr (N.D.K.)

^3^ Laboratoire Matière et Systèmes Complexes, UMR 7057 CNRS Université Denis Diderot Paris-VII, Bâtiment Condorcet, 10 rue Alice Domon et Léonie Duquet, 75205 Paris, France; evdokia.oikonomou@univ-paris-diderot.fr (E.K.O.)

***** Correspondence: e-mail; j.kallitsis@upatras.gr; Tel.: +30-261-096-2952; Fax: +30-261-099-7122

Received: date; Accepted: date; Published: date


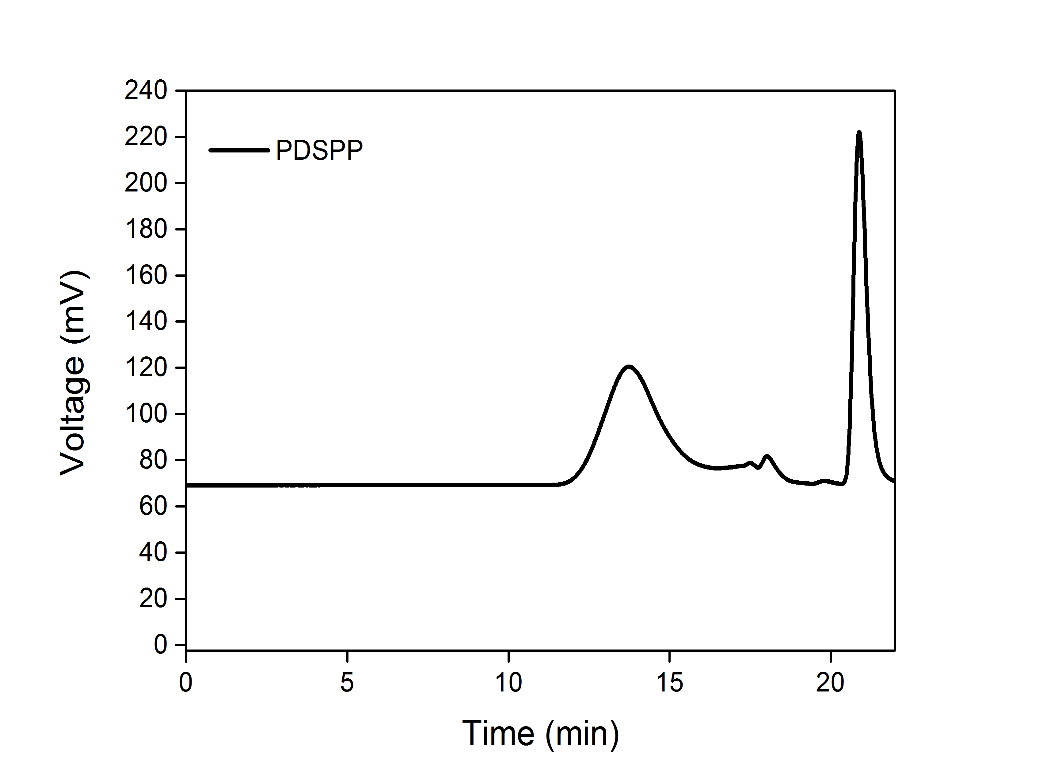


**Figure S1.** GPC chromatograph of the PDSPP homopolymer.


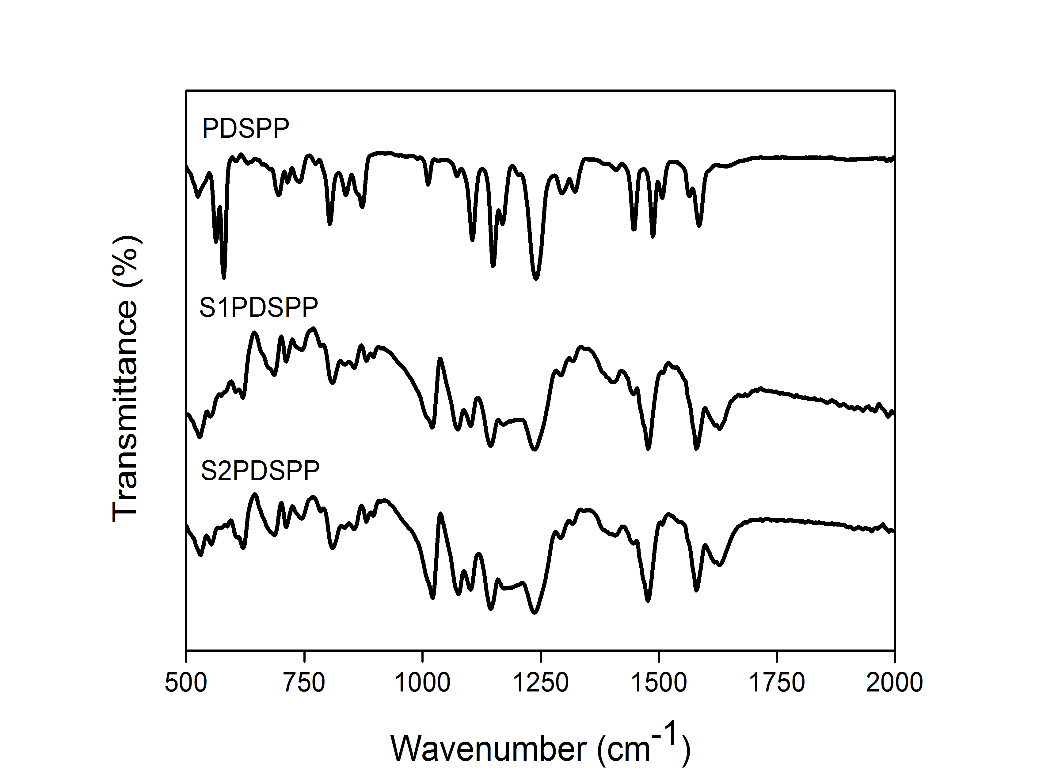


**Figure S2.** ATR-FTIR spectra of the PDSPP and SPDSPP homopolymers.


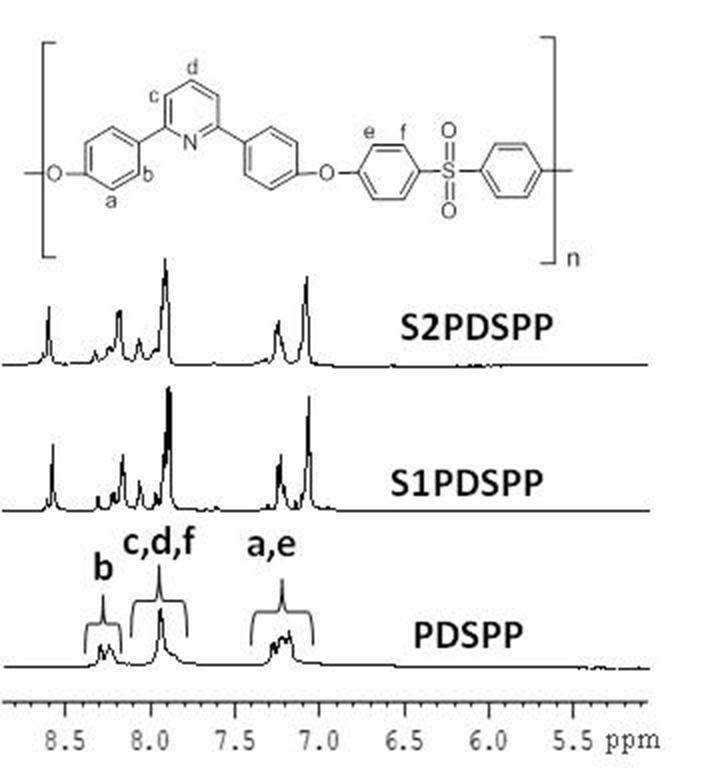


**Figure S3.** ^1^H NMR spectra of the PDSPP and SPDSPP homopolymers.


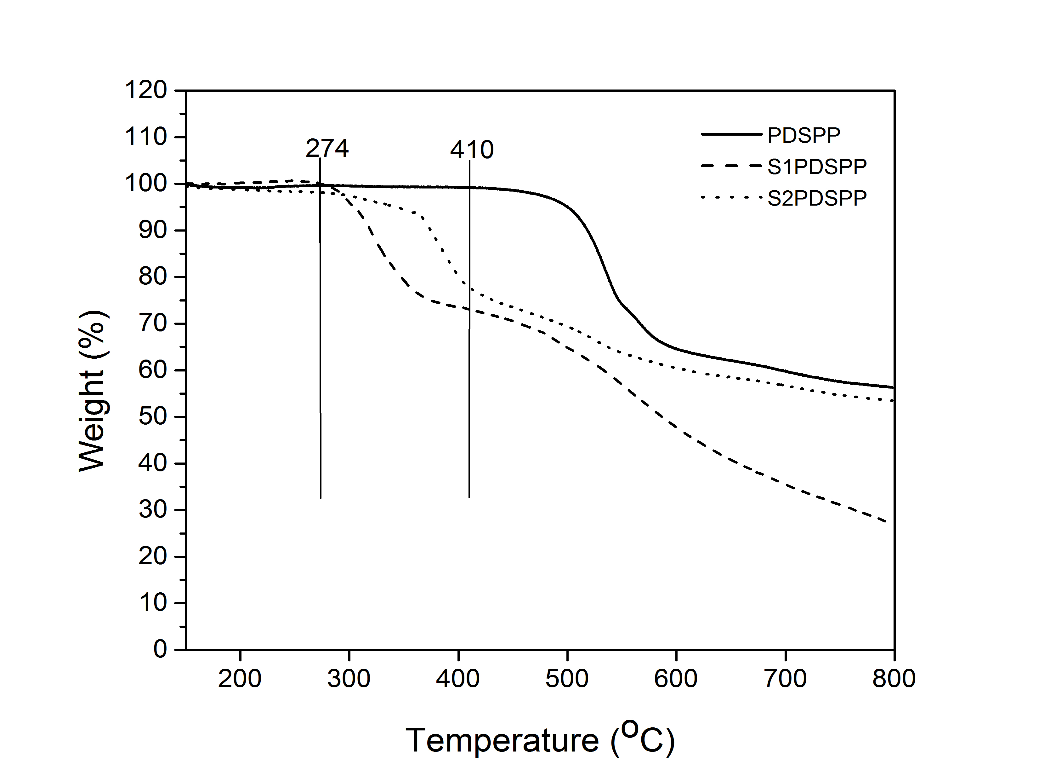


**Figure S4.** TGA curves of the PDSPP and SPDSPP homopolymers.

**Table S1.** Quantities, along with Mn, Mw and PDI as calculated from the GPC characterization for the PDSPP homopolymers.

| Polymer | Quantity (g) | Mn | Mw | PDI |
| --- | --- | --- | --- | --- |
| PDSPP1 | 5 | 47000 | 68000 | 1.43 |
| PDSPP2 | 7.5 | 51000 | 98000 | 1.92 |
| PDSPP3 | 5 | 71000 | 125000 | 1.75 |

**Table S2.** DS (mol%) estimated from the ^1^H-NMR and TGA techniques, as well as IEC for the S1PDSPP and S2PDSPP homopolymers.

| Polymer | DS (mol%) from ^1^H-NMR | DS (mol%) from TGA | IEC (meq/g) |
| --- | --- | --- | --- |
| S1PDSPP | 175 | 195 | 1.38 |
| S2PDSPP | 150 | 135 | 2.56 |

**Table S3.** Preparation of membranes with 90/10 w/w PDSPP/SPDSPP in DMA, at 5% or 15% w/w, casting at 80 °C until 35%, 50%, 75%, 90% w/w total final polymers’ concentration and immersion in H_2_O at 25 °C or 60 °C for 3 h.

| Membrane | PDSPP initial/final concentration (w/w%) | SPDSPP initial/final concentration (w/w%) | Initial/final concentration (w/w%) in DMA | Temperature (°C)/Coagulation time (h) in H_2_O | Membrane thickness (μm) |
| --- | --- | --- | --- | --- | --- |
| M1’-35D | 4.5/31.5 | 0.5/3.5 | 95/65 | 25/3 | - |
| M1’-50D | 4.5/45 | 0.5/5 | 95/50 | 25/3 | - |
| M1’-75D | 4.5/67.5 | 0.5/7.5 | 95/25 | 25/3 | 25 |
| M1’-90D | 4.5/81 | 0.5/9 | 95/10 | 25/3 | 25 |
| M1’-35DT | 4.5/31.5 | 0.5/3.5 | 85/65 | 60/3 | - |
| M1’-50DT | 4.5/45 | 0.5/5 | 85/50 | 60/3 | - |
| M1’-75DT | 4.5/67.5 | 0.5/7.5 | 85/25 | 60/3 | 30 |
| M1’-90DT | 4.5/81 | 0.5/9 | 85/10 | 60/3 | 30 |
