# Supplementary figures and images for: Preparation of Porous Polymeric Membranes Based on a Pyridine Containing Aromatic Polyether Sulfone
Source: Polymers (Basel). 2019 Jan 2;11(1):59. doi: 10.3390/polym11010059 (PMC6402226; doi:10.3390/polym11010059)

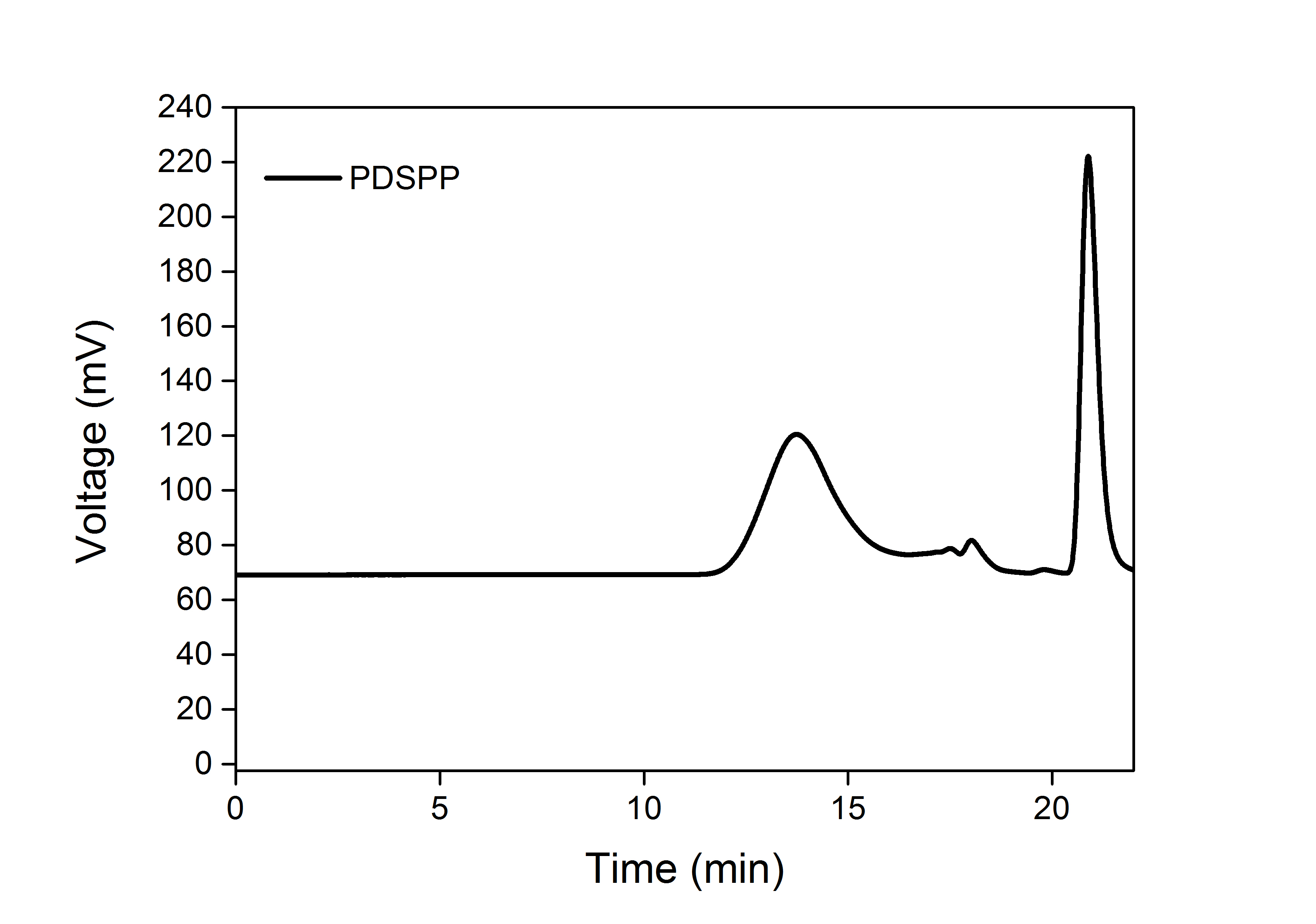

Supplement: Supplementary file 1 [file polymers-11-00059-s001.zip › polymers-403298-Supplementary Materials/Figure S1.tif]

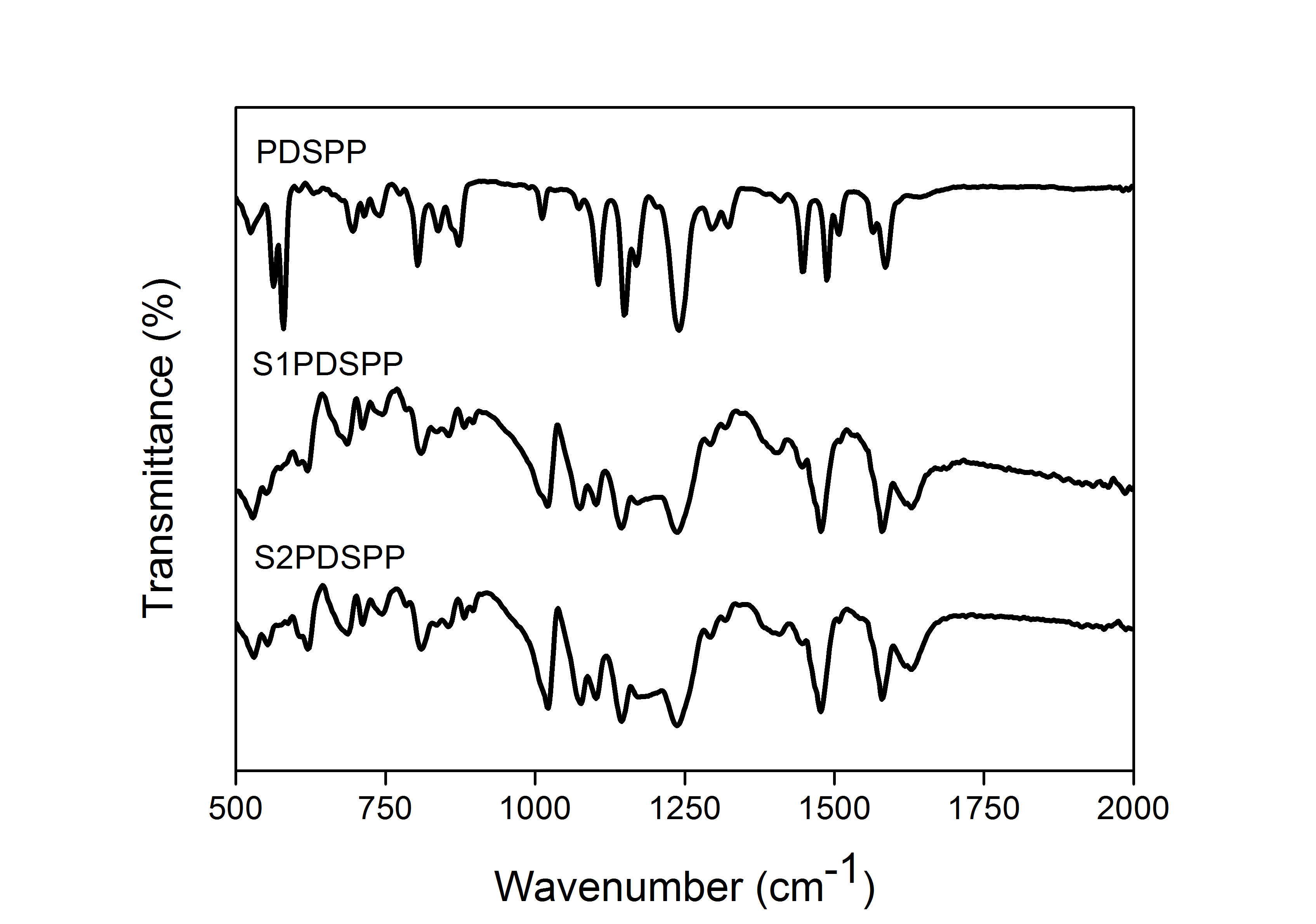

Supplement: Supplementary file 1 [file polymers-11-00059-s001.zip › polymers-403298-Supplementary Materials/Figure S2.tif]

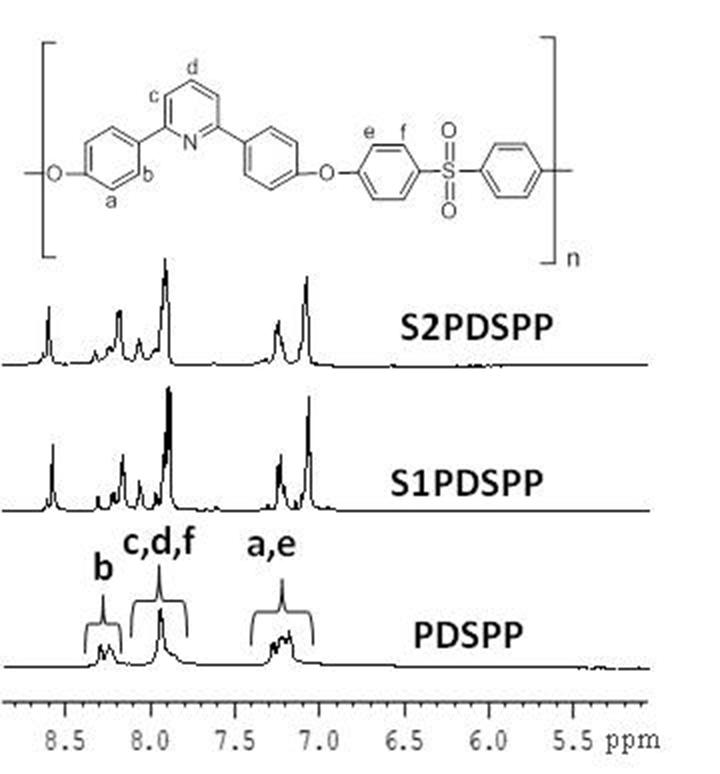

Supplement: Supplementary file 1 [file polymers-11-00059-s001.zip › polymers-403298-Supplementary Materials/Figure S3.tif]

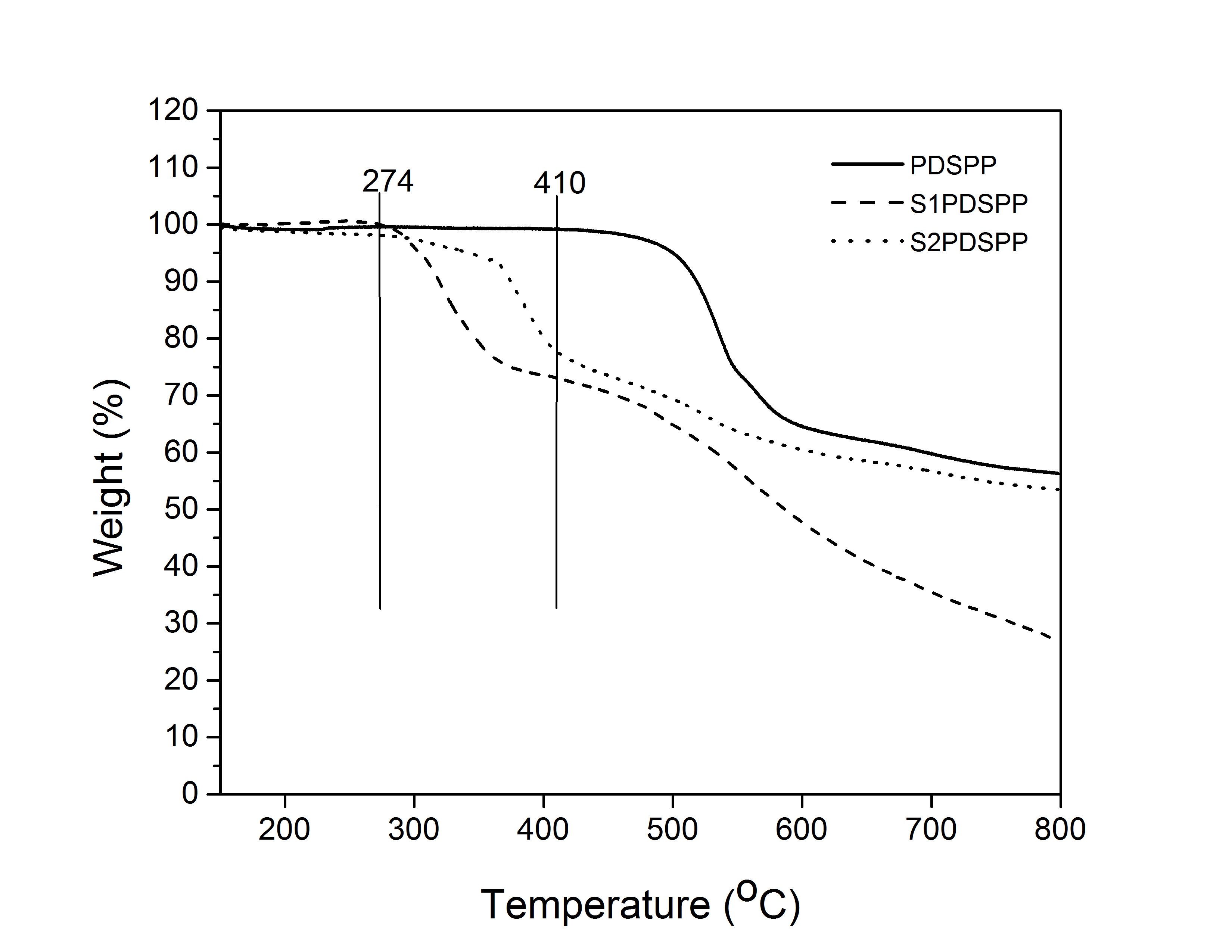

Supplement: Supplementary file 1 [file polymers-11-00059-s001.zip › polymers-403298-Supplementary Materials/Figure S4.tif]
